# Persistent Quantitative Vitality of Stem Cell Graft Is Necessary for Stabilization of Functional Brain Networks After Stroke

**DOI:** 10.3389/fneur.2019.00335

**Published:** 2019-04-05

**Authors:** Claudia Green, Anuka Minassian, Stefanie Vogel, Michael Diedenhofen, Dirk Wiedermann, Mathias Hoehn

**Affiliations:** ^1^In-vivo-NMR Laboratory, Max Planck Institute for Metabolism Research, Cologne, Germany; ^2^Department of Radiology, Leiden University Medical Center, Leiden, Netherlands; ^3^Percuros B.V., Enschede, Netherlands

**Keywords:** stem cell graft, graft vitality, stroke, functional neuronal networks, resting state fMRI, bioluminescence imaging

## Abstract

Stem cell treatment after stroke has demonstrated substantial outcome improvement. However, monitoring of stem cell fate *in vivo* is still challenging and not routinely performed, yet important to quantify the role of the implanted stem cells on lesion improvement; in several studies even mortality of the graft has been reported. Resting state functional magnetic resonance imaging (rs-fMRI) is a highly sensitive imaging modality to monitor the brain-wide functional network alterations of many brain diseases *in vivo*. We monitor for 3 months the functional connectivity changes after intracortical stem cell engraftment in large, cortico-striatal (*n* = 9), and in small, striatal (*n* = 6) ischemic lesions in the mouse brain with non-invasive rs-fMRI on a 9.4T preclinical MRi scanner with GE-EPI sequence. Graft vitality is continuously recorded by bioluminescence imaging (BLI) roughly every 2 weeks after implantation of 300 k neural stem cells. In cortico-striatal lesions, the lesion extension induces graft vitality loss, in consequence leading to a parallel decrease of functional connectivity strength after a few weeks. In small, striatal lesions, the graft vitality is preserved for the whole observation period and the functional connectivity is stabilized at values as in the pre-stroke situation. But even here, at the end of the observation period of 3 months, the functional connectivity strength is found to decrease despite preserved graft vitality. We conclude that quantitative graft viability is a necessary but not sufficient criterion for functional neuronal network stabilization after stroke. Future studies with even longer time periods after stroke induction will need to identify additional players which have negative influence on the functional brain networks.

## Introduction

Ischemic stroke is the third most common cause of death and the leading cause for disabilities worldwide (1). There is still no clinically accepted therapy available except for the FDA-approved thrombolysis, which is only available to a small proportion of patients due to a limited time window for treatment and serious risks for side effects (2). Therefore, new therapeutic approaches are urgently required. With the enormous progress in stem cell biology during the past several years application of stem cells for regenerative therapy has rapidly gained high interest. Several experimental studies on rodent models of stroke have reported outcome improvements after stem cell implantations (3–8). Most of these studies focused on the stem cell effect on lesion volume or relied on behavioral assays for the read-out of outcome improvement (7–11). Other reports concentrated mainly on the structural and functional integration of the stem cell grafts in the host tissue (12, 13).

A novel approach based on resting state functional magnetic resonance imaging (rs-fMRI) accesses the functional networks of the whole brain and thereby allows to reveal functional network alterations during brain diseases (14–17). Applying rs-fMRI to stroke we (18) and others (19–21) have reported on the whole-brain network deficits after stroke, characterized by substantial decrease of the functional connectivity strength and by the far reaching extension of this decrease to both hemispheres, irrespective of localization of the ischemic tissue.

In the first report on response of functional networks to stem cell implantation after induction of large cortico-striatal ischemic lesion we described an early paracrine effect (18). This paracrine effect was reflected by the early and complete stabilization of the functional connectivity by the stem cell graft. However, in that report, this stabilization was lost after a few weeks when the graft viability was substantially decreasing by ~70% of its original level (18), as monitored by bioluminescence imaging (BLI).

In the present investigation we compare functional networks in large cortico-striatal lesions, in correspondence with our previous study (18), with those of animals with lesion extension limited to the striatum. This comparison is of specific interest, as it can provide insight on the effect of later expansion of the ischemic territory on the cortical graft. We hypothesized that graft vitality in the striatal lesion group will persist long-term and that in consequence the functional connectivity stabilization will continue, thus leading to an enduring therapeutic effect. To that aim, we monitored in all animals the graft viability by BLI (22–24) and repetitively recorded rs-fMRI up to 3 months after stroke induction. We compared the results of both groups, with small striatal and with large cortico-striatal lesions, and looked for connections between graft viability and successful functional connectivity stabilization for therapeutic assessment.

## Materials and Methods

### Experimental Animals and Experimental Design

All animal experiments were carried out in accordance with the guidelines of the German Animal Welfare Act and approved by the local authorities (Landesamt für Natur, Umwelt und Verbraucherschutz Nordrhein-Westfalen). Animals were socially housed under a fixed 12:12 h light/darkness cycle with *ad libitum* access to food and water.

A total of 15 adult, male NMRI-*Foxnl*^nu/nu^ mice (12–14 weeks old, 32–37 g; Janvier, France) were used in this study. All surgical experiments were performed under anesthesia with 1.5–2% Isoflurane in a mixture of 70/30% N_2_O/O_2_ atmosphere. Analgetic treatment included subcutaneous injection of 4 mg/kg Carprofen (Rimadyl, Zoetis, Berlin, Germany) twice a day, for 3 days following surgery. Two days after stroke induction by the filament occlusion method (18, 23), the animals received an intracortical stem cell implantation on the ipsilateral hemisphere. Resting state fMRI was performed 1 week before, as well as 1, 2, 4, 8, and 12 weeks after stroke induction and cell implantation (2 days after stroke induction), while vitality of the transgenic graft was monitored by BLI at weeks 2, 3, 4, 5, 7, 9, 10, and 12 after stroke.

### Transient Middle Cerebral Artery Occlusion

After MRI baseline measurements, focal ischemic stroke was induced by transient occlusion of the middle cerebral artery (MCA) with an intraluminal filament as described previously (23, 25). In brief, mice were anesthetized with Isoflurane and the right common carotid artery (CCA), the external and the internal carotid artery (ICA) were exposed. A rubber-coated filament (length of 20 mm, diameter of 170 μm at the tip; Doccol Corp., Sharon, USA) was inserted into the ICA until blockage of the MCA. After 30 min occlusion time, the filament was removed and the CCA was ligated permanently. Analgesic medication was started directly before surgery and further provided during 3 days thereafter. Location and size of the ischemic damage was assessed at 48 h after stroke induction by MRI.

### Intracortical Implantation and Monitoring of Transgenic Neural Stem Cells

The commercially available human neural stem cell line H9-NSC (WA09, Life Technologies) was lentivirally transduced with the construct EF1α-Luc2-T2A-eGFP to express the light emitting imaging reporter firefly luciferase (*Luc2*) and an enhanced green fluorescence protein (*eGFP*) under the constitutive promotor of the elongation factor 1 alpha (EF1α), as described earlier (26).

Two days after stroke induction and before cell implantation, all animals received a T2-weighted MRI scan to determine successful stroke induction and, based on MRI results, were divided into cortico-striatal and striatal lesion group, respectively. For cell implantation, NMRI-*Foxnl*^nu/nu^ mice were anesthetized and the head was fixed in a stereotaxic frame (Stoelting, Dublin, Ireland). A small hole was drilled into the skull above the planned injection site, and a Hamilton syringe (26 G needle) was slowly inserted ipsilesionally into the somatosensory cortex (S1) at the injection site with AP: +0.5 mm, DV: −0.5 mm according to bregma. The lateral coordinate had previously been selected individually based on the respective T2-weighted MRI at 48 h and was set to implant the cells adjacent to but outside the lesion for each individual animal, at approximately L: 1–2 mm.

Nine animals which had a cortico-striatal stroke and six animals with a striatal stroke were injected with a total of 2 μl of H9-EF1α-Luc2-T2A-eGFP cells at a concentration of 150,000 cells/μl, resuspended in Hank's balanced salt solution (HBSS, Life Technologies), at a flow rate of 150 nl/min. After injection, the needle was kept in place for 5 min before withdrawal.

Cell vitality was monitored via BLI, which was recorded once every 1–2 weeks for the duration of the study. For BLI measurements the mice were intraperitoneally injected with 300 mg/kg D-Luciferin sodium salt (Synchem, Felsberg, Germany) solved in Dulbecco's phosphate-buffered saline (Life Technologies) and subsequently anesthetized with a mixture of 2% Isoflurane in 70/30% N_2_O/O_2_ atmosphere (24). Photon Emission (PE) was recorded for 30 min with the Photon Imager IVIS SPECTRUM CT (Perkin-Elmer, Waltham, MA, USA) under Isoflurane anesthesia.

### Structural and Functional MRI Data Acquisition

All experiments were conducted on a small animal 9.4 T horizontal MRI system with a 20 cm bore diameter and actively shielded gradient coils (BGA12S2, >660 mT/m, Bruker BioSpin, Ettlingen, Germany). RF excitation and signal reception were performed with a 1H quadrature cryogenic surface coil (CryoProbe, Bruker BioSpin). Image acquisition was executed with ParaVision 5.1 software (Bruker BioSpin GmbH). Physiological parameters were monitored with the SA Instruments 1025T System (SA Instruments, NY, USA) and recorded with DASYlab Software (Measurement Computing, Norton, USA). Body temperature was measured via a fiber optic rectal probe (SA Instruments, NY, USA) and kept constant at 37°C ± 1.0°C by a water circulating system (medres, Cologne, Germany). Animals were anesthetized with 2% Isoflurane in a mixture of 70/30% N_2_/O_2_ and the head was fixated in the animal cradle with ear bars and a tooth bar in a nose cone with continuous gas flow. At the beginning of the imaging session, Isoflurane was reduced to 1.5%.

Functional activity was acquired as resting state functional MRI. All MRI sessions were preceded by a three-plane scout scan (Tripilot), adjustment of the RF signal receiver gain, and a FieldMap with consecutive local shim to optimize magnetic field homogeneity and image quality. An anatomical reference scan was acquired with a T2-weighted TurboRARE sequence with a field of view (FOV) of 17.5 × 17.5 mm^2^, 48 contiguous slices of 0.2 mm slice thickness, matrix dimension of 256 × 256, repetition time (TR) = 5,500 ms, echo time (TE) = 32.5 ms, and a RARE factor of 8 with two averages. Moreover, a T2-weighted spin echo sequence was recorded at 48 h after stroke induction, directly before the stem cell grafting, with equivalent parameters.

A bolus of 0.1 mg/kg Medetomidine (Domitor®, Elanco), suspended in 250 μl NaCl, was administered subcutaneously 15–20 min prior to the functional imaging scan, with subsequent reduction of Isoflurane level to 0.5%, following an earlier reported protocol (18, 27). A gradient-echo echo-planar imaging (GE-EPI) sequence was used for rs-fMRI with the following parameters: FOV: 17.5 × 17.5 mm^2^, matrix size: 96 × 96, in-plane resolution: 182 × 182 μm^2^, TR = 2,840 ms, and TE = 18 ms. One hundred and five image sets were acquired with 16 slices each, with slice thickness of 0.5 mm and inter-slice gap of 0.1 mm, recorded non-interleaved and covering the whole forebrain, starting only after a minimal time of 10 min on reduced Isoflurane levels.

### rs-fMRI Data Processing

All datasets were brain extracted, slice-wise motion corrected with FSL (FMRIB Software Library; http://www.fmrib.ox.ac.uk/fsl) and linearly detrended (28). To allow for comparison of different imaging sessions for analysis of specific Regions of Interest (ROIs), we co-registered our datasets to publicly available mouse brain atlases of the whole brain (29) and the neocortex (30) through the following multi-step process: (1) First, an in-house made MRI mouse brain template was warped to both brain atlases with FSL. (2) The acquired anatomical reference scan from each individual session was linearly co-registered to the in-house mouse brain template and (3) the functional datasets to the anatomical datasets via rigid-body transformation. (4) Finally, the co-registration results themselves were discarded but the transformation matrices from all steps were concatenated, inverted, and then applied to the mouse brain atlas to fit the atlas to the individual original data, thus avoiding deformation bias of the raw data. Six ROIs, or nodes, were extracted from the sensorimotor network, separate for each hemisphere: The primary and secondary motor cortex (M1/M2), the primary somatosensory cortex excluding the limb regions (S1 w/o limbs), the S1 fore- and hindpaw limb regions (S1 limbs), the secondary somatosensory cortex (S2), the thalamus (Th), and the caudate putamen (CPu) (Supplementary Figure 1). Bilateral ROIs were only analyzed until week 2 after stroke induction for accuracy purposes of ROI extraction. For longer periods after stroke, due to brain swelling and/or atrophy, only the contralateral hemisphere was analyzed.

Regression of physiological noise was performed by regressing out the recorded respiratory signals, motion parameters, and drifts up to the second order (28). Furthermore, the data were spatially smoothed in-plane with a Gaussian filter of FWHM = 0.3 mm. To limit contributions from low-frequency signal drifts and high-frequency physiological noise, the time series signal was bandpass-filtered to 0.01–0.08 Hz and normalized. Group-wise full Pearson correlation between pairs of ROIs (i.e., nodes) of the average time series was calculated with FSLNets (v0.6; www.fmrib.ox.ac.uk/fsl) and compiled in matrix form. All correlation values were transformed to *z*-values by taking the Fisher transformation of the *r*-values prior to averaging.

### Statistical Analysis

Statistical analysis was performed with IBM SPSS 24 software (IBM Corporation, New York, NY, USA), Matlab 2014b (The MathWorks Inc., Natick, MA, USA) and GraphPad Prism v.8.0.2 for Windows (GraphPad Software, La Jolla California USA). Statistical significance levels were set to ^*^p < 0.05, ^**^p < 0.005.

#### Functional Connectivity

Univariate repeated measures ANOVA was used for the statistical analysis of the cross-correlation, within-group results and mixed ANOVA for the cross-correlation between group effects with *post-hoc* Bonferroni corrections, respectively. Most of the comparisons did not survive the most conservative statistical correction for multiple comparisons. We refrained from the less accurate statistical analysis but decided to present the changes over time and the difference between groups as strong trends, unless where statistical significance is explicitly given.

#### Bioluminescence Imaging

A mixed-effects model with restricted maximum likelihood was applied to the BLI data to calculate statistical within-group effects of consecutive time points. The alpha level was set to 0.05, a Greenhouse-Geisser correction was enabled for non-spherical data and a Sidak's *post-hoc* test corrected for multiple comparison.

## Results

### Ischemic Lesion Types

**Figure 1:**
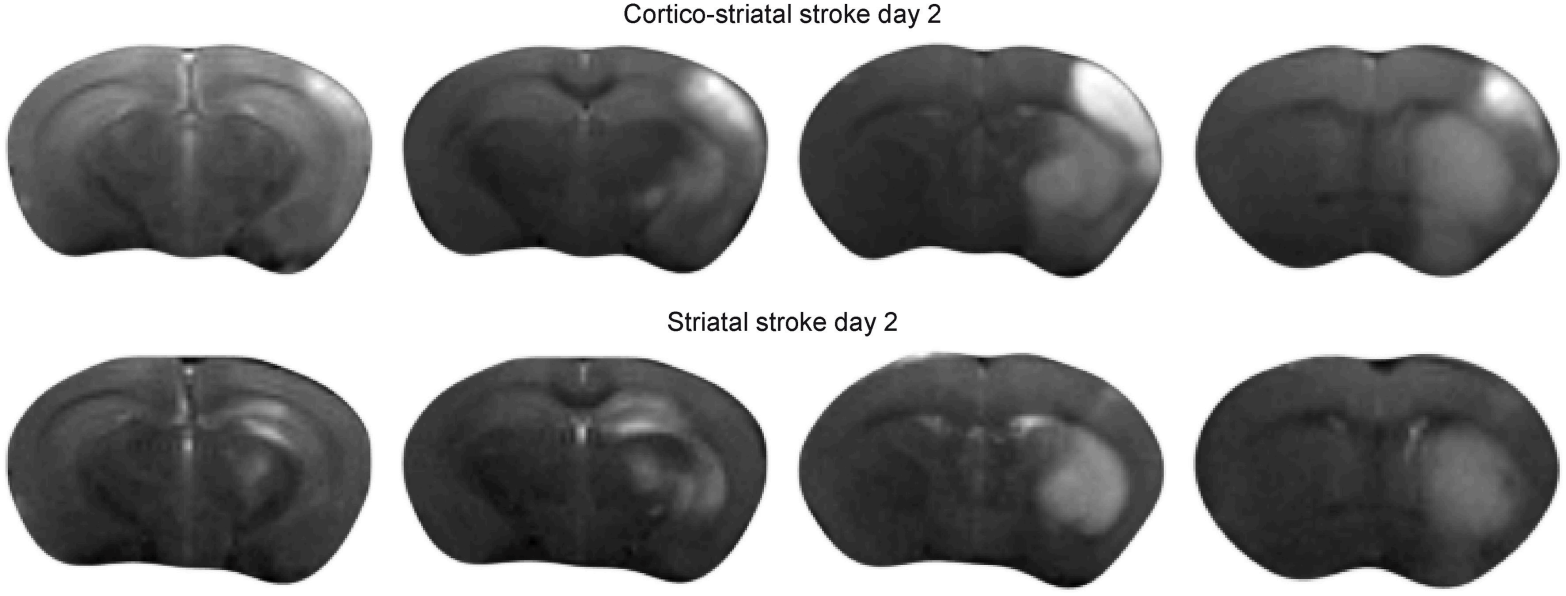
Acute ischemic stroke lesion on T2-weighted MRI. Multislice T2-weighted MR images at 48 h after stroke induction, directly before implantation of neural stem cells. The hyperintensity on the MR images clearly demarcates the extent of the ischemia-affected tissue: the upper row shows the spread of the lesion across the cortical and sub-cortical tissue areas of a representative animal with cortico-striatal lesion. The lower row presents the situation for a representative mouse of the striatal lesion group.

Forty eight hours after intraluminal filament occlusion of the MCA, all animals received T2-weighted MRI to confirm successful stroke induction. Two types of ischemic lesions were found on the MRI: a small lesion restricted to the striatum (*n* = 6), and a large lesion encompassing the striatum plus a large part of the cortex (*n* = 9). Figure 1 presents the ischemic lesion on T2-weighted MRI of a representative mouse of both lesion types.

### Vitality of the Stem Cell Graft During 12 Weeks

**Figure 2:**
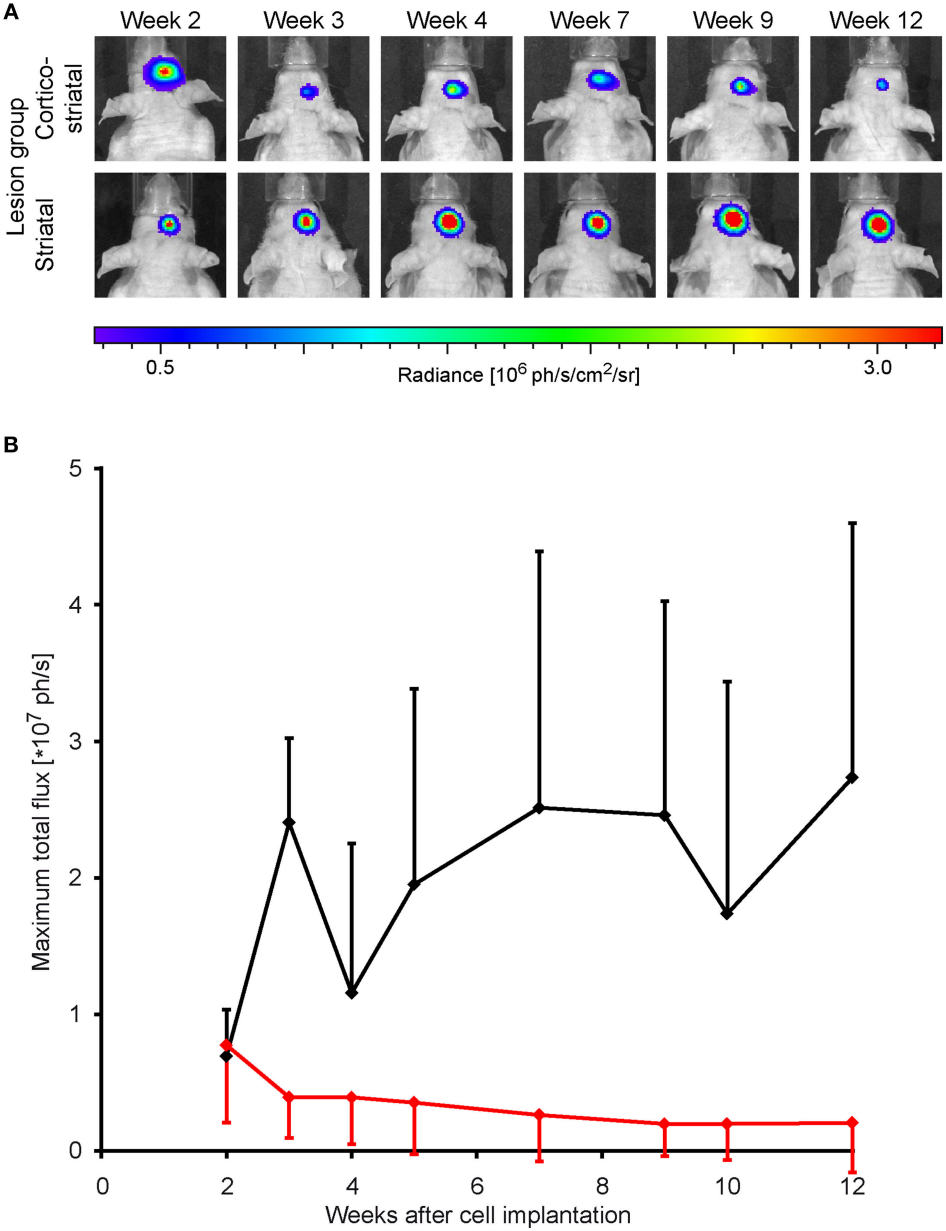
Stem cell graft viability during 3 months after implantation. Bioluminescence imaging of the luciferase-expressing neural stem cells was recorded over 3 months every 1 or 2 weeks after implantation into the cortex of the ischemic hemisphere. **(A)** Representative animals of the cortico-striatal and striatal lesion group at various time points after implantation. The BLI intensity of the cortico-striatal lesion animal shows a continuous decrease over time, while the BLI intensity of the mouse with striatal lesion remains stable over the whole 3 months. **(B)** Quantitative analysis of BLI intensity of both lesion groups with in-group standard deviations: the striatal lesion group (black line) shows an overall stable BLI signal intensity on the pre-stroke level. The BLI signal intensity of the cortico-striatal group (red line) presents a rapid decrease with time, leveling off at ~30% of the pre-stroke value.

Forty eight hours after stroke induction, 300,000 neural stem cells, previously transduced to express luciferase under constitutive control, were implanted into the S1 cortex. BLI was performed every 1–2 weeks, starting at week 2 post MCA occlusion. At the time point two weeks after implantation, BLI signal intensity was strong and indistinguishable between both lesion groups. In the group of animals with striatal lesion, BLI signal remained high with little non-significant [*F*_(7, 30)_ = 4.02] changes throughout the 12 weeks observation. In the cortico-striatal lesion group, BLI signal started to decrease, non-significantly [*F*_(7, 59)_ = 0.67] between consecutive weeks, shortly after 2 weeks after cell implantation (Figure 2A). The quantitative analysis of the BLI signal intensity of the cell grafts is given in Figure 2B, clearly showing the persistent high level of stable graft viability for the striatal lesion group (black line). The strong signal decrease in the cortico-striatal lesion group to approximately 30% on average of the original average value indicated a vitality loss of a large graft fraction within the first weeks after implantation (red line).

### Functional Neuronal Network Changes

#### Both Hemispheres During the First 2 Weeks After Stroke

**Figure 3:**
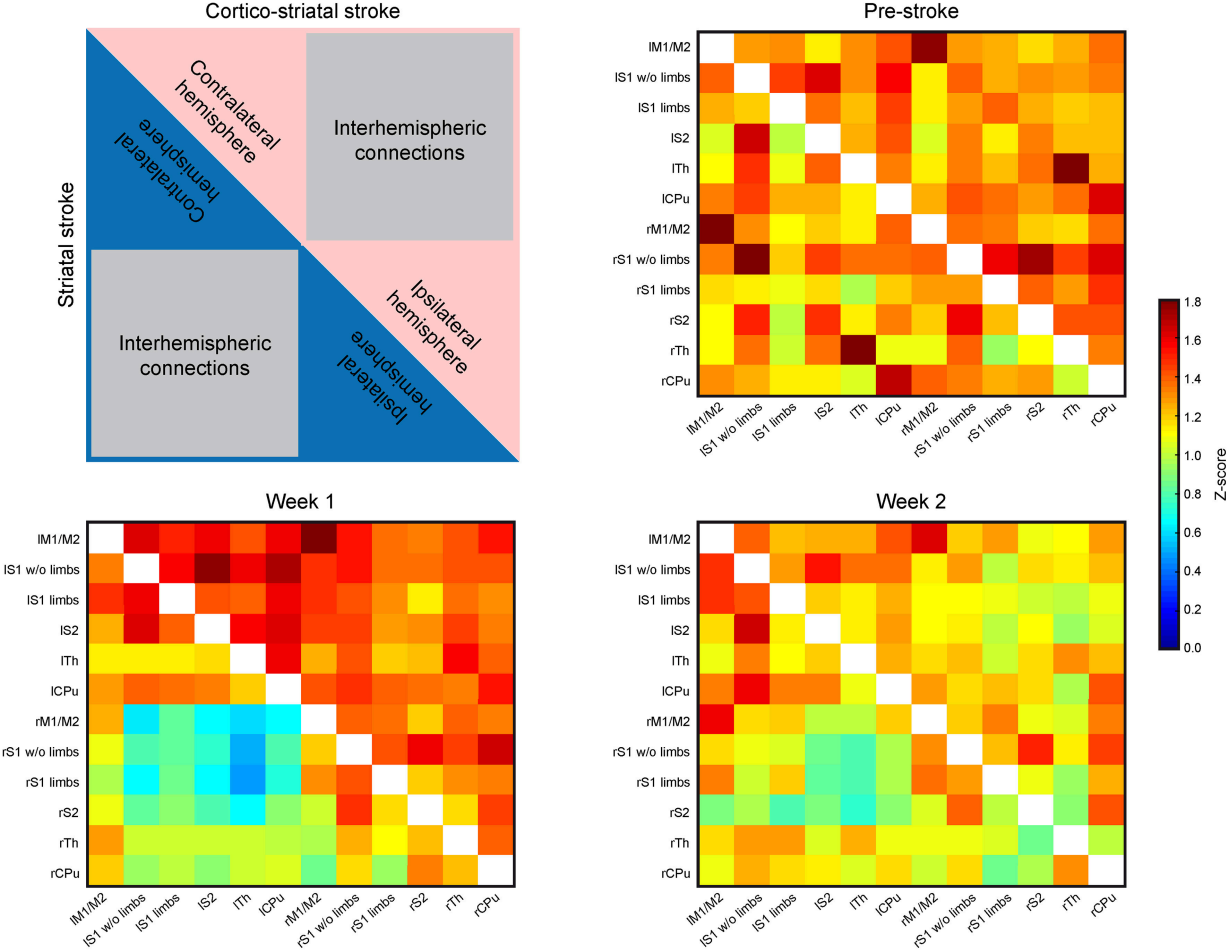
Functional connectivity matrices of the whole brain. The matrices contain the connectivity values (*z*-score) of selected brain regions. The distributions of values within the matrices are laid out in the schematic at the upper left. The upper, red triangle stands for the cortico-striatal group, the lower, blue triangle stands for the striatal group. The regions representing *intra*-hemispheric connectivities of ipsi- and contralateral hemisphere, and the *inter*-hemispheric connectivities are marked. The three matrices show the ipsi- and contralateral brain connectivity values at pre-stroke time (upper right), at week 1 post-stroke (lower left) and at week 2 post-stroke induction (lower right). During the control time point (pre-stroke) both groups show closely equal connectivity strength across the whole brain (upper right). At week 1 post-stroke induction, the cortico-striatal group shows a slight hyperconnectivity, while the connectivity values of the striatal group remain unaffected by stroke induction, with the exception of a small field of reduced connectivities within the *inter*-hemispheric connections. At week 2 post-stroke (lower right), both groups show values close to the pre-stroke condition in the *intra*-hemispheric parts. The *inter*-hemispheric parts show a reduction relative to the pre-stroke condition.

Presentation of the functional connectivity strength of the sensorimotor networks in matrix form is schematically presented in Figure 3 (top left), with values of both lesion groups combined in one matrix to better visualize the differences between them. Before stroke induction, the functional connectivity values (*z*-score values; Figure 3, top right) show equal correlation strength in both hemispheres within each lesion group, as presented in the two triangular segments of the matrix triangle for the respective group. Furthermore, comparison of the matrices of both groups does not show statistically significant differences before stroke induction (Figure 3, top right). At week 1 after stroke induction, a small, but non-significant increase of connectivity strength is noted in the cortico-striatal group across the whole brain, irrespective of the hemispheric side (Figure 3, bottom left; upper matrix triangle), while the functional connectivity values remained widely unchanged in the striatal group (Figure 3 bottom left; lower matrix triangle). Interestingly, in the striatal group, the *intra*-hemispheric cross-correlations of both hemispheres remained indistinguishable among each other and from pre-stroke condition, while parts of the *inter*-hemispheric cross-correlations show a clearly demarcated rectangle with reduced values (Figure 3, bottom left, blue field in lower matrix triangle). After 2 weeks, the functional connectivity values have renormalized in both groups, with the *inter*-hemispheric connectivity field with reduced values of the striatal group being less pronounced and showing a clear trend toward normalization (Figure 3, bottom right). Interestingly, of all six calculated interhemispheric homotopic connectivties, thalamic connectivity decreases the most in both groups, with an even higher reduction in the striatal group (−76% vs. −40.9% for the cortico-striatal group).

#### The Contralateral Hemisphere During 12 Weeks After Stroke

**Figure 4:**
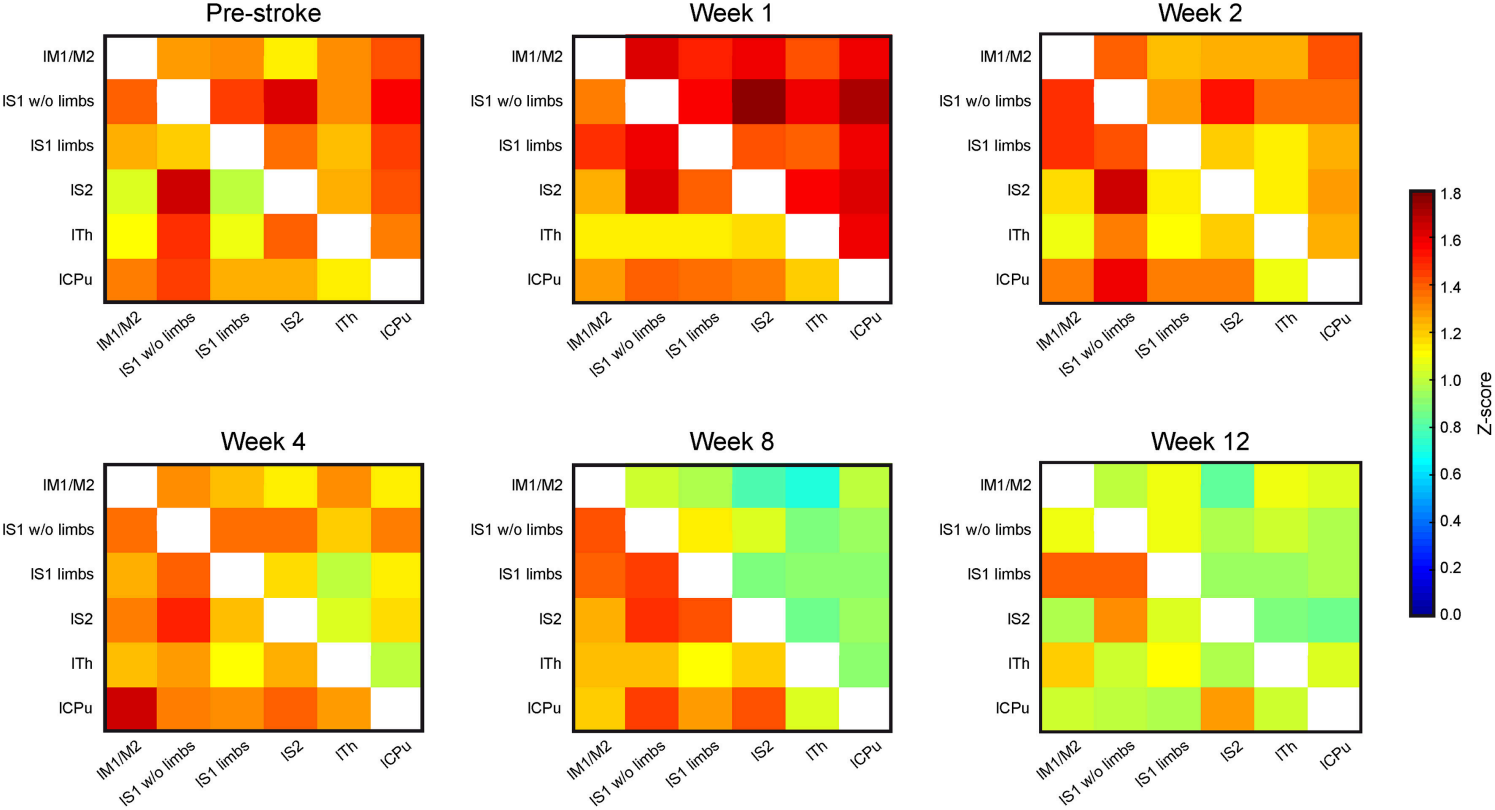
Connectivity matrices of the contralesional hemisphere over 3 months. The connectivity matrix of the contralateral hemisphere for the cortico-striatal group in the upper triangle, and for the striatal group in the lower triangle, with data presented for pre-stroke, weeks 1, 2, 4, 8, and 12 post stroke induction and neural stem cell implantation. The *z*-score values of the sensorimotor functional network of the cortico-striatal lesion group present a connectivity decrease, being substantial from week 4 on, and reaching its minimum at week 8. In the striatal lesion group, *z*-score values remain without clear changes to the pre-stroke situation during the first 8 weeks. Only at the last time point, at week 12 post implantation, the connectivity values decrease approximating those of the cortico-striatal group.

Brain swelling followed by later brain atrophy prohibited the reliable co-registration of the ipsilateral, ischemic hemisphere with the assigned regions of the anatomic brain atlas to determine corresponding functional connectivities at times later than 2 weeks post stroke induction. Therefore, the connectivity matrices of the sensorimotor networks were limited to the healthy hemisphere after week 2 (Figure 4). The matrices of the contralateral hemisphere up to week 2 are already included in the whole brain matrices in Figure 3. Analysis of the functional connectivity changes on the ipsilateral hemisphere during the chronic phase, though also highly interesting, will have to await future development of more complex co-registration strategies of the rs-fMRI data with the mouse brain atlas, assuring correct anatomical alignment under severe brain deformations.

The functional connectivity strength of the cortico-striatal group at week 2 is still closely similar to the pre-stroke situation. However, a substantial reduction of connectivity strength is noted beginning at week 4. This reduction becomes very pronounced at week 8, remaining at similarly low values at week 12 (Figure 4; upper matrix triangles) with the strongest reduction noted for the connection between the primary somatosensory cortex wo limbs and the secondary somatosensory cortex (−67.2% reduction in z-score value between pre-stroke and 12 weeks post MCA occlusion), as well as the caudate putamen with the S1 wo limbs (−59.1%) and S2 (−66.9%).

On the contrary, in the striatal group functional connectivity values remain high until week 8, similar to the pre-stroke situation. Only in week 12, this group shows distinctly lowered connectivity strengths, approximating the state of low cross-correlations of the cortico-striatal group. For the striatal group highest differences are observed between the lS1 wo limbs with the thalamus (−44.9% from before stroke, to 12 weeks after), and with the caudate putamen (−46.7%). Some correlations in the striatal group even increase slightly between pre-stroke measurement and the last time point. This is specifically observed for anatomically adjacent regions, such as the lS1 limbs and lS1 wo limbs (+13.4%), and the lM with the lS1 limbs (+11%). On average the functional connectivity strength measured in *z*-score correlation coefficient reduces between the pre-stroke situation and 12 weeks after stroke induction for the cortico-striatal group by 39.4 ± 15.3%, whilst the striatal-only group only shows a reduction by 15.0 ± 21.4% averaged over all connection pairs.

#### Scatterplot Analysis of Functional Connectivity Changes Over Time

**Figure 5:**
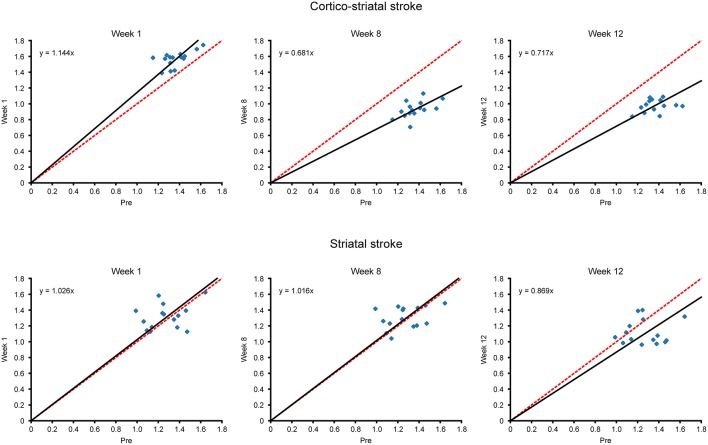
Scatterplots of matrix elements between two time points. The *z*-score values of all connectivities of the contralateral hemisphere, contained in the matrices of Figure 4, are compared for different time points with their respective values before stroke induction, as presented in scatter plot diagrams. Deviation of the scatterplots from the identity line is presented for the two time points in each diagram. The identity line indicating no changes for the individual matrix elements between two time points is marked in red, while the fit through the scatter plot and passing through zero is given in black. The slope of the fit, in deviation from slope = 1.000 for the identity line, indicates the overall increase or decrease of the *z*-score values from the pre-stroke time point to the second selected time point. For the cortico-striatal group (upper row diagrams), the diagram at week 1 shows the slight slope increase (slope_wk1; cortico−striatal_ = 1.144) for the hyperconnectivity; at weeks 8 and 12, the slopes have substantially decreased (slope_wk8; cortico−striatal_ = 0.681, slope_wk12; cortico−striatal_ = 0.717), demonstrating the weakening of the sensorimotor functional network at those times. For the striatal group (lower row diagrams), complete superposition with the identity line is preserved for week 1 and also at the late chronic period at week 8, indicating complete stabilization of the pre-stroke connectivity strength by the neural stem cells (slope_wk1; striatal_ = 1.026, slope_wk8; striatal_ = 1.016). Only at week 12, here the slope has noticeably decreased to slope_wk12_ = 0.869, which, however, remains still above the slope of the cortico-striatal group at this time point.

Additional to the matrix presentation of individual correlations between specific ROIs above, we also aimed to analyze the overall functional differences over time after stem cell grafting and stroke. Scatter plots were generated plotting all functional connectivity matrix elements at the pre-stroke condition against the ones of three time points after grafting. The values at week 1 were chosen for the acute time window and the values at weeks 8 and 12, respectively, for the late changes. In the case of no changes of the individual matrix elements between two time points, all matrix elements would be expected to lie on the central diagonal through zero with a slope of 1 (identity line). Deviation of the fitted slope from the central diagonal (slope = 1.000, red line in Figure 5) hereby indicates the overall increase or decrease of the *z*-score values from the pre-stroke condition to the selected second time point.

In the cortico-striatal lesion group (Figure 5, upper row) the slope at week 1 is only slightly increased (slope_wk1; cortico−striatal_ = 1.144) indicating little change from the baseline condition. At weeks 8 and 12, the slope has substantially deviated from the identity diagonal, with strongly decreased values (slope_wk8; cortico−striatal_ = 0.681; slope_wk12; cortico−striatal_ = 0.717).

In contrast, for the striatal lesion group (Figure 5, lower row), the slope at week 1 and still at week 8 is indistinguishable from the identity diagonal (slope_week1;striatal_ = 1.026; slope_week8;striatal_ = 1.016). Only at the end of the monitoring period, at week 12, the slope has decreased from the identity diagonal, reaching a low value at slope_week12;striatal_ = 0.869. However, this deviation from the identity diagonal remained still less than the corresponding value of the cortico-striatal lesion group.

## Discussion

We have combined the two imaging modalities, resting state functional MRI and BLI to (i) observe modulations of the functional neuronal network changes after stroke by stem cell grafts and to (ii) monitor the viability of the stem cells during 3 months following stroke induction. Our present results indicate that stabilization of the functional connectivity of the sensorimotor networks after stroke requires persistent and quantitative viability of the stem cell graft.

### Quantitative Determination of Graft Survival

Many investigations have been concerned with functional improvement of the brain after stem cell implantations (4–6, 12, 13, 31). They mostly relied on behavioral evaluations of the functional deficit and improvement, while histological analysis was used for demonstration of existing stem cell grafts and their potential neuronal differentiation (7–11, 32). However, these histological studies are commonly qualitative assessments of the existence of implanted stem cells, without quantification of the fraction of stem cells survived at the end of the observation period and in correspondence to the functional status. This means that those studies missed a clear temporal relationship between the functional improvement and the persistent survival of the graft. This is, however, of high importance as several studies had reported high mortality of engrafted stem cells (33–39). Rather than on qualitative histological assessment, here, we have relied on the well-established quantitative BLI as a tool to quantitatively determine cell viability. As the firefly luciferase which we used as imaging reporter to follow vitality of our engrafted stem cells depends on the presence of ATP and oxygen for the photon generation, the BLI signal intensity is directly proportional to the number of luciferase positive stem cells (40). It should be noted though, that in an earlier report, we had described differentiation of the exact same stem cells, used in the present study, into neurons 3 months after implantation into the corticostriatal lesion model reporting HuNu/NeuN double-positive cells in a qualitative immunohistochemical analysis (18).

We have demonstrated that correct placement of the graft, sufficiently distant to the ischemic lesion, allows long-term and fully quantitative preservation of the graft viability. However, in the case of our cortico-striatal stroke lesion, the stem cells had been implanted too close to the acute lesion rim, resulting in a later substantial loss of viability as the lesion expansion jeopardized the survival of the stem cell majority (18). This was demonstrated by the severe reduction of BLI signal intensity of the stem cell graft. In contrast, in the case of the striatal lesions, the cortical graft preserved its early full level of vitality over the 3 months observation time, as demonstrated with the steady BLI signal over 3 months following engraftment. This finding is in full agreement with earlier reports of the same stem cell line, H9, in NMRI-*Foxnl*^nu/nu^ mice. There, a stable quantitative preservation of cortical graft viability in the healthy mouse brain (26, 41) and also in a different cortical stroke model (41) was seen with robust and stable BLI signal intensity for 7 weeks (26) and even for 3 months (41).

### Functional Connectivity of the Sensorimotor Networks

Persistent strong reduction of the functional connectivity strength of both hemispheres had been shown by us (18) and others (19–21) for stroke situation without cell treatment. In correspondence with our earlier study (18), we found a transient stabilization of the functional connectivity after cortical stem cell engraftment in animals with large cortico-striatal lesions. The stabilization remained for ~4 weeks when the connectivity strength decreased to approach that of animals with stroke only. Those reductions in functional connectivity affected also the contralateral hemisphere showing the massive far-range effect of the stroke lesion on the neuronal networks. In contrast, our data on the striatal lesions showed a robust stabilization of the functional connectivity by the stem cell graft, remaining stable beyond the 2 month time point, and still being stronger at week 12 than in the case of cortico-striatal lesions. Interestingly, also the selective intermittent inter-hemispheric connectivity decrease, seen in the striatal lesions with stem cells, showed a clear tendency for re-normalization within 2 weeks.

### Correlation Between Graft Vitality and Functional Network Stabilization

In the large cortico-striatal lesions, the lesion expanded into the adjacent graft location, thereby jeopardizing the graft vitality. When the graft vitality is reduced, the connectivity stabilization is weakened in consequence and is finally lost, here after about 4 weeks. However, some cells were still surviving and had been shown earlier to differentiate into neurons (18). In contrast, in the small striatal lesion, the graft is not threatened by expansion of the ischemic territory into the cortical cell location and vitality of the graft remains stable over the whole observation period of 12 weeks. In consequence, functional connectivity strength remains at the level of control before stroke at least till week 8. Surprisingly, at week 12 the connectivity strength is also substantially weakened in the striatal lesion group, approximating the lower values of the cortico-striatal group—despite persistent vitality. From our findings we conclude that vitality of the stem cell graft is a necessary, but not a sufficient condition for stabilization of functional neuronal networks.

From our present results it becomes plausible that full quantitative graft vitality is required for functional connectivity stabilization. Previously, we had concluded from comparison of the functional connectivity strength of animals after stroke with and without stem cell engraftment that a paracrine effect must be the responsible mechanism for the stem cell mediated functional network stabilization (18). The here observed much delayed functional connectivity weakening at week 12 post-stroke induction and engraftment points to additional effects that further influence the functional networks stability. Late rejection of the graft can be excluded because of persistent high BLI signal intensities. However, interaction of the graft with other mechanisms must be sought that modulate the paracrine effect, which is therapeutically active during the first 2 months. Here, a hot candidate with time dependent activity are the immune cells of which a strong interaction with stem cells has been discussed (42). Microglia and macrophages have been shown to be of anti-inflammatory, protective polarization phenotype in the acute stroke phase and switch to a pro-inflammatory, aggravating polarization phenotype at later time points (43). Although little is known about the immune cells' activity in the chronic phase of 3 months, it may be speculated that the immune cell activity may affect the paracrine effect of the graft by modulating the stem cell mediated secretion of cytokines and growth factors. Then, external modulation of the polarization state of the immune cells, which has previously been demonstrated to have a protective neuronal effect (44, 45) may also contribute to the recovery of the graft's therapeutically active paracrine effect. Future studies may follow this interaction between stem cell graft and immune cells by multicolor BLI (46), allowing simultaneous monitoring of the graft viability and of the immune cell polarization (47), while assessing the effect of polarization status modulation on the functional brain networks over time.

## Conclusions

Our present investigations on functional connectivity after stroke have contributed further evidence for a paracrine effect of stem cells stabilizing the functional neuronal networks already directly after implantation. Further, we have shown here that this paracrine effect of the stem cells requires quantitative preservation of graft vitality. Finally, the quantitative conservation of vitality is a necessary, but not a sufficient condition for the long-term therapeutic effect of functional network stabilization. Additional influencers, indicated to become of increasing importance in the chronic phase, such as immune cell activity, will need to be analyzed in future for complete and enduring preservation of functional networks.

## Ethics Statement

All animal experiments were carried out in accordance with the guidelines of the German Animal Welfare Act and approved by the local authorities (Landesamt für Natur, Umwelt, und Verbraucherschutz Nordrhein-Westfalen).

## Author Contributions

CG designed the study, performed the MRI experiments, and wrote the manuscript. AM performed the infarct surgery. SV analyzed the BLI experiments. MD performed data analysis of the rs-fMRI data. DW analyzed the rs-fMRI data and wrote the manuscript. MH designed the study, analyzed the data, and wrote the manuscript.

### Conflict of Interest Statement

MH was employed part time by Percuros B.V. The remaining authors declare that the research was conducted in the absence of any commercial or financial relationships that could be construed as a potential conflict of interest.

## Supplementary Material

Supplementary Figure 1 Overlay of regions of interest on MR images. The six regions analyzed on each hemisphere for analysis of functional network changes are overlaid on coronal RARE MR images of a representative healthy mouse brain. This was achieved by co-registration of the mouse brain atlas with the MRI mouse brain template which again was co-registered with the individual RARE MRI data set. This procedure is described in detail in the Methods section. The four coronal images presented here cover the range of the mouse brain for all relevant sensorimotor cortex areas and the thalamus, at the rostral-caudal axis, equidistantly from −1.55 mm to +1.05 mm relative to bregma.
