# Supplementary figures and images for: Persistent Quantitative Vitality of Stem Cell Graft Is Necessary for Stabilization of Functional Brain Networks After Stroke
Source: Front Neurol. 2019 Apr 5;10:335. doi: 10.3389/fneur.2019.00335 (PMC6460358; doi:10.3389/fneur.2019.00335)

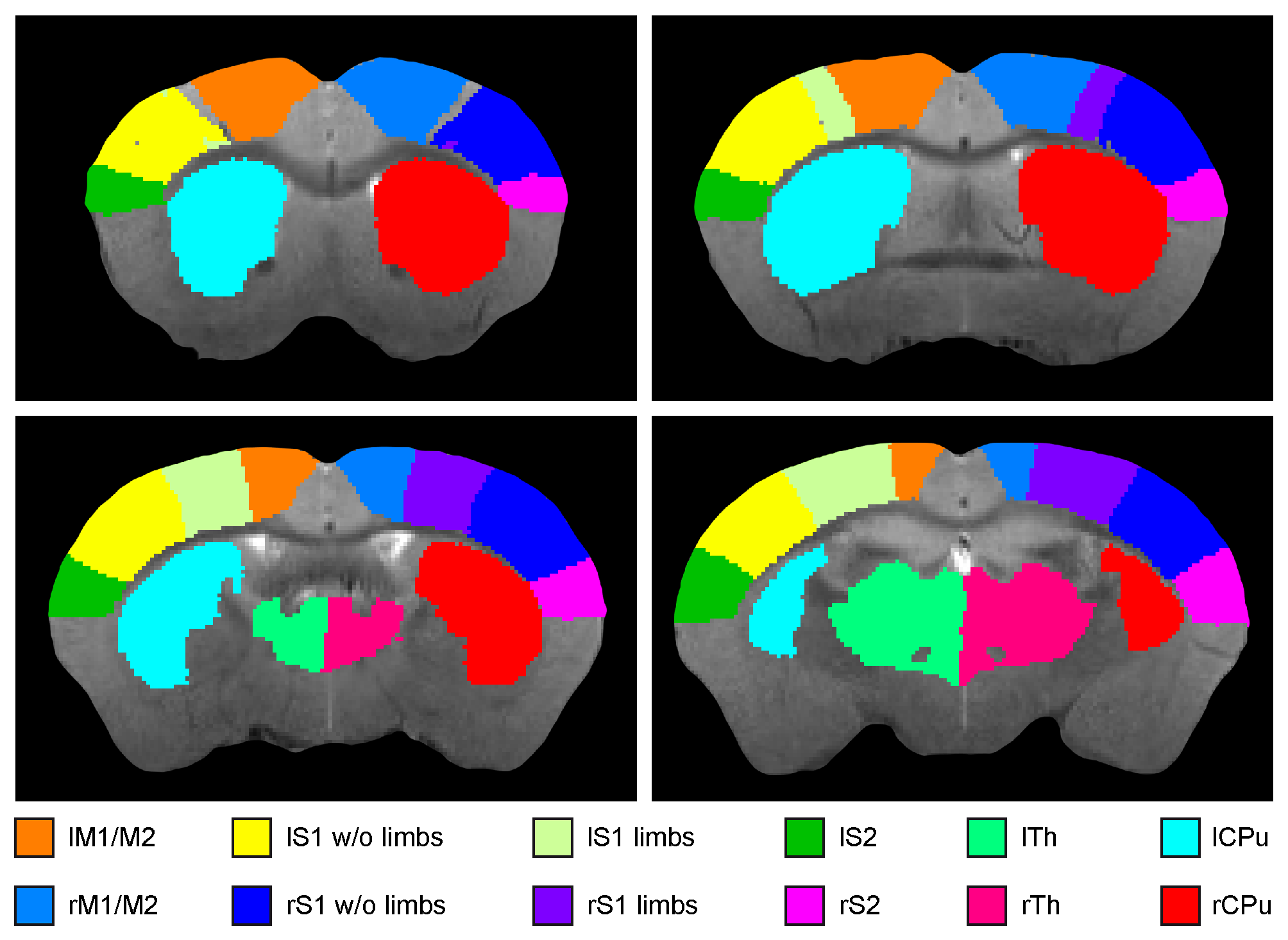

Supplement: Supplementary Figure 1 — Overlay of regions of interest on MR images. The six regions analyzed on each hemisphere for analysis of functional network changes are overlaid on coronal RARE MR images of a representative healthy mouse brain. This was achieved by co-registration of the mouse brain atlas with the MRI mouse brain template which again was co-registered with the individual RARE MRI data set. This procedure is described in detail in the Methods section. The four coronal images presented here cover the range of the mouse brain for all relevant sensorimotor cortex areas and the thalamus, at the rostral-caudal axis, equidistantly from −1.55 mm to +1.05 mm relative to bregma. [file Image_1.TIF]
